# Acid Degradation, Structure Characterization of a Novel Polysaccharide from Leaves of *Isatis indigotica* Fort. with Immunomodulatory Activity

**DOI:** 10.3390/molecules31091461

**Published:** 2026-04-28

**Authors:** Yu Shen, Xuefeng Wang, Huiming Zhang, Yuliang Wang, Zheng Wang, Yiying Zhang, Hongbo Zhao

**Affiliations:** 1 College of Pharmacy, Jiamusi University, Jiamusi 154007, China; shenyu@jmsu.edu.cn (Y.S.); 238163035@stu.jmsu.edu.cn (X.W.); wangyuliang@jmsu.edu.cn (Y.W.); 258073057@stu.jmsu.edu.cn (Z.W.); 2 College of Basic Medical Sciences, Jiamusi University, Jiamusi 154007, China; zhmjms@jmsu.edu.cn; 3 Department of Epidemiology and Biostatistics, School of Public Health, Jiamusi University, Jiamusi 154007, China; 4 College of Rehabilitation Medicine, Jiamusi University, Jiamusi 154007, China

**Keywords:** Isatidis Folium polysaccharides, acid degradation, structural characterization, immunomodulatory activity

## Abstract

The immunomodulatory potential of natural polysaccharides is often limited by their structural complexity and high molecular weight. In this study, DFIP-A3-1 (*M*_w_ = 8.68 × 10^3^ g/mol) was obtained from the leaves of *Isatis indigotica* Fort. by ultrafiltration, DEAE-650M, and Sephadex G-100 chromatography, followed by acid degradation. Fortunately, DFIP-A3-1 exhibited the most potent immunostimulatory activity in vitro. HPGPC, HPSEC-MALLS-RID, GC-MS, FT-IR, Congo red tests, SEM, and AFM were used to characterize their structure, and 1D/2D NMR was used for further investigation of DFIP-A3-1 for in-depth structural clarification. DFIP-A3-1 was primarily composed of Rha and Gal. Based on methylation and NMR analyses, the structure of DFIP-A3-1 was elucidated as follows: →1)-β-Galp-(4→1,4)-α-Rhap-(2→1,4)-α-Rhap-(2→1)-β-Galp-(6→1)-β-Galp-(6→1,6)-β-Galp-(3→1,6)-β-Galp-(3→. Furthermore, DFIP-A3-1 was found to exhibit a triple-helix conformation. DFIP-A3-1 markedly upregulated the secretion of NO, IL-6, and TNF-α and enhanced the mRNA expression levels of their related genes in RAW 264.7 cells. Moreover, DFIP-A3-1 activated p-IκBα, p-p65, and TLR4, while co-treatment with TAK-242 markedly suppressed the expression of these pathway-related proteins. All of the aforementioned findings suggested that DFIP-A3-1 is a promising natural immunomodulatory drug deserving of additional research and use.

## 1. Introduction

The immune system uses effective recognition and response mechanisms to act as the body’s initial line of defense. It continuously monitors for foreign pathogens and abnormal cells, while also clearing senescent or dead debris. This intricate process is essential for preserving physiological homeostasis and health [1]. Immune deficiency predisposes individuals to recurrent infections and increased tumor risk, whereas immune hyperactivation or dysregulation may result in autoimmune diseases, allergies, and chronic inflammatory disorders [2]. Thus, normal immune regulation is critical for health maintenance [3]. Immunomodulation can be achieved through various approaches, primarily involving the intervention of chemical drugs, biological agents, and natural bioactive compounds. The underlying mechanisms frequently include immune cell activation, manipulation of cytokine release, and control of important signaling pathways such as the NF-κB and MAPKs signaling pathways. Among these, natural bioactive compounds exhibit unique advantages and broad application prospects due to their multi-target characteristics and favorable biocompatibility. They have a lot of opportunities for preserving immunological homeostasis and for creating functional foods or supplemental medications [4,5,6,7].

Isatidis Folium (also known in Chinese as Da-Qing-Ye) is the dried leaves of the plant *Isatis indigotica* Fort. (Brassicaceae). It is extensively cultivated in northern China, North Korea, and Japan. Isatidis Folium is a type of traditional medicinal herb that has been used to cure aphthous stomatitis, fever with headache, sore throats, and jaundice [8]. Contemporary pharmacological studies indicate that Isatidis Folium and its extracts exhibit good biological activities such as antiviral, immunomodulatory, antioxidant and lipid-lowering [9,10,11]. Isatidis Folium contains diverse chemical components, including polysaccharides, saponins, and flavonoids [12]. Polysaccharides are most likely an important ingredient in the efficacy of Isatidis Folium [13]. Up until now, polysaccharides have been shown by abundant studies to possess low toxicity, high safety, and significant biological activity [14]. This has resulted in an increasing number of applications for polysaccharides; hence, it is essential to comprehend their structure in order to properly research their applications [15,16]. Numerous important biological functions, such as immunomodulatory, anticancer, antioxidant, and anti-inflammatory properties, are known to be displayed by polysaccharides [17,18,19,20,21]. However, research on the polysaccharides of Isatidis Folium has primarily focused on their anti-inflammatory and antioxidant properties, while in-depth investigations into the relationship between their structure and immunomodulatory activity remain limited.

Molecular weight can be regarded as a more direct indicator of the immunological activity of polysaccharides, even if other parameters like monosaccharide content and glycosidic linkages also play a role. It was reported that *Glycyrrhiza* polysaccharide (molecular weight: 6.5 kDa), *Tremella fuciformis* polysaccharide (3 kDa), and marine fungus *Aureobasidium melanogenum* polysaccharide (8 kDa) exhibited superior immunomodulatory activity [22,23,24]. Therefore, to better utilize the biological activity of polysaccharides, their molecular weight can be altered through degradation. Current methods for polysaccharide degradation include physical, chemical, and biological approaches. Acid hydrolysis, in particular, offers a rapid and efficient means of reducing molecular weight. The key advantages of acid degradation include high efficiency, rapid processing, operational simplicity, and suitability for large-scale production [25]. This reduced the molecular weight of the macromolecular polysaccharide polymers, which enabled them to bind more effectively with specific receptors on cell membranes. However, a thorough explanation of the connection between the biological activity of degraded polysaccharides and their structural alterations is still lacking. Additionally, the purity and structure of polysaccharides have a major impact on the immune regulatory function of cells [26,27]. Therefore, polysaccharides with well-defined structures and high purity are essential for clinical application.

In the present work, a combination of purification strategies and analytical methods was employed to extract polysaccharides from the leaves of *Isatis indigotica* Fort. These fractions were subsequently subjected to acid hydrolysis using trifluoroacetic acid. Through comprehensive analytical investigations, the structural characteristics of these bioactive polysaccharides were thoroughly explored, which provided data support for the further development and application of Isatidis Folium. Furthermore, by examining the immunological activity of the degraded polysaccharides, the correlation between their structure and function was revealed. This discovery is essential for evaluating these polysaccharides’ potential as innovative immunotherapeutic agents, which lays the foundation for the development and application of new immunological health products.

## 2. Results and Discussion

### 2.1. Extraction, Isolation and Purification of FIP

**Figure 1:**
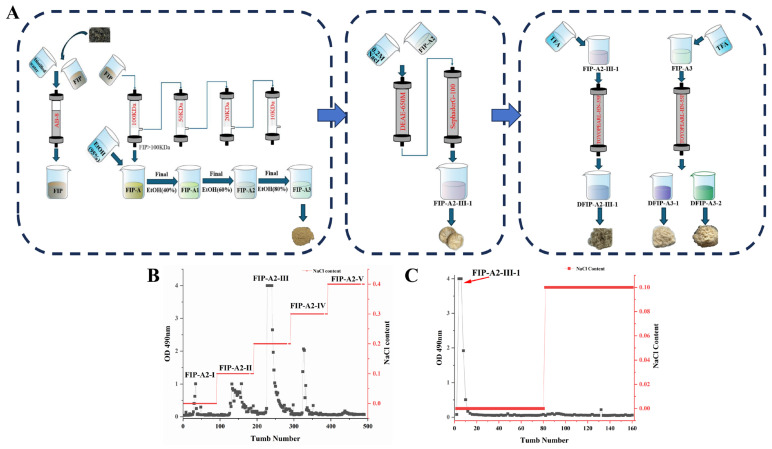
(**A**) Workflow for isolation and purification of polysaccharides from leaves of *Isatis indigotica* Fort. (**B**) Elution curves of FIP-A2-I~V components. (**C**) Elution curves of FIP-A2-III-1.

A yield of 15.03% (w/w) of the crude polysaccharides from *Isatis indigotica* Fort. leaves was obtained by hot water extraction followed by ethanol precipitation. Subsequently, Folium isatidis polysaccharide (FIP) was obtained using AB-8 macroporous adsorbent resin for further decolorization and deproteinization. Subsequently, five sub-fractions—designated as FIP-A (13.7%, w/w), FIP-B (1.8%, w/w), FIP-C (0.4%, w/w), FIP-D (0.1%, w/w), and FIP-E (84.0%, w/w)—were obtained by sequential ultrafiltration of FIP through membranes with molecular weight cut-offs of 100, 50, 20, and 10 kDa. FIP-A was then subjected to ethanol fractional precipitation by adjusting the ethanol concentration to 40%, 60%, and 80%. The resulting sub-fractions were named FIP-A1 (8.2%, wt%), FIP-A2 (49.2%, wt%) and FIP-A3 (42.6%, wt%), respectively. FIP-A2 on a DEAE-650M column produced five subfractions, designated FIP-A2-I through FIP-A2-V. FIP-A2-III was further purified by gel filtration on a Sephadex G-100 column (GE Healthcare Life Sciences, Uppsala, Sweden), which yielded a major subfraction named FIP-A2-III-1 with a yield of 49.27% (w/w). The separation and preparation flowchart and the elution curve are shown in Figure 1.

### 2.2. Acid Hydrolysis of FIP-A2-III-1 and FIP-A3

Acid hydrolysis of FIP-A2-III-1 and FIP-A3 was performed with 0.5 M trifluoroacetic acid (TFA) at 80 °C for 2 h. As shown in Figure S1, following separation and purification on an HW-55F column and preparation using a Waters 515-2414 system, the pooled fractions were freeze-dried to yield DFIP-A2-III-1 (15.8%, wt%), DFIP-A3-1 (22.5%, wt%), and DFIP-A3-2 (2.8%, wt%), respectively.

### 2.3. Determination of Homogeneity and Molecular Weight

As shown in Figure S2, high performance gel permeation chromatography—evaporative light scattering detector (HPGPC-ELSD) analysis revealed that FIP-A2-III-1, FIP-A3, DFIP-A2-III-1, DFIP-A3-1, and DFIP-A3-2 all exhibited a single symmetrical peak, indicating that they are homogeneous polysaccharides.

**Figure 2:**
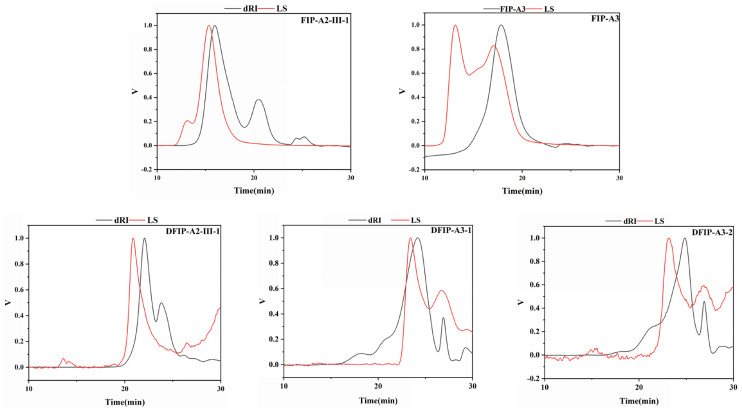
HPSEC-MALLS-RID chromatogram of FIP-A2-III-1, FIP-A3, DFIP-A2-III-1, DFIP-A3-1, and DFIP-A3-2.

Figure 2 and Table 1 summarize the molecular weight data obtained by high performance size exclusion chromatography with multi-angle laser light scattering and refractive index detector (HPSEC-MALLS-RID). The *M*_w_ (molecular weight) of FIP-A2-III-1 decreased from 2.29 × 10^5^ g/mol before degradation to 5.11 × 10^3^ g/mol after degradation (DFIP-A2-III-1). Similarly, the molecular weight of FIP-A3 decreased from 9.85 × 10^4^ g/mol to 8.68 × 10^3^ g/mol (DFIP-A3-1) and 2.24 × 10^3^ g/mol (DFIP-A3-2), respectively. These results indicated a significant reduction in molecular weight after degradation. Additionally, the polydispersity indices (*M*_w_/*M*_n_) of the five polysaccharide samples were measured as 1.16, 1.43, 1.09, 1.54, and 1.28, corresponding to FIP-A2-III-1, DFIP-A2-III-1, FIP-A3, DFIP-A3-1, and DFIP-A3-2, respectively. The restricted molecular weight distribution of the polysaccharides was further supported by these observations. For example, the molecular weight of *Belamcanda chinensis* polysaccharide decreased from 2.62 × 10^5^ g/mol to 6.25 × 10^4^ g/mol after TFA [28]. Upon TFA hydrolysis, the molecular weight of the polysaccharide derived from Hericium erinaceus was reduced to 1.57–1.97 × 10^6^ Da [29]. These results collectively confirm that acid degradation is effective in regulating the molecular weight of polysaccharides.

### 2.4. FT-IR Analysis

**Figure 3:**
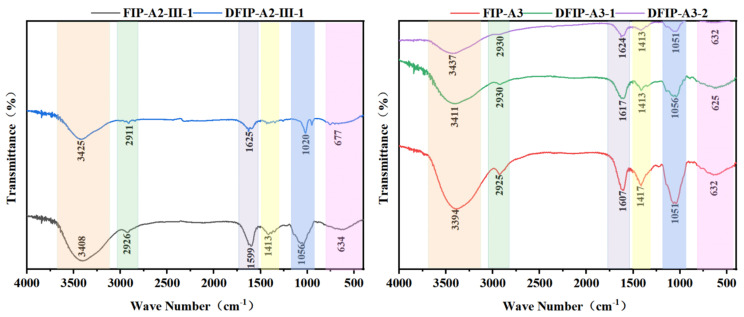
The FT-IR spectra of FIP-A2-III-1, DFIP-A2-III-1, FIP-A3, DFIP-A3-1 and DFIP-A3-2.

The polysaccharide functional groups were successfully characterized through analysis by Fourier transform infrared (FT-IR) spectroscopy (Figure 3). In the range of 3000–3500 cm^−1^, an obvious absorption peak characteristic of O–H stretching vibrations was detected [30]. Absorption bands corresponding to C–H and C=O stretching vibrations were detected at approximately 2900 cm^−1^ and 1700 cm^−1^ [31]. Furthermore, an absorption peak attributed to the C–H bending mode was recorded at approximately 1400 cm^−1^ [32]. An absorption signal corresponding to the C–O stretching vibration was recorded at approximately 1100 cm^−1^ [33]. In addition, a weak signal detected within the 520–820 cm^−1^ region indicated the existence of β-glycosidic bonds [34]. The presence of these absorption peaks was taken as an indication of the polysaccharide components. High similarity was observed among the absorption peaks of FIP-A2-III-1, DFIP-A2-III-1, FIP-A3, DFIP-A3-1, and DFIP-A3-2. Based on the absence of characteristic C=O stretching signals from their carboxyl groups, these polysaccharides were identified as neutral [35].

### 2.5. Monosaccharide Composition Analysis

**Figure 4:**
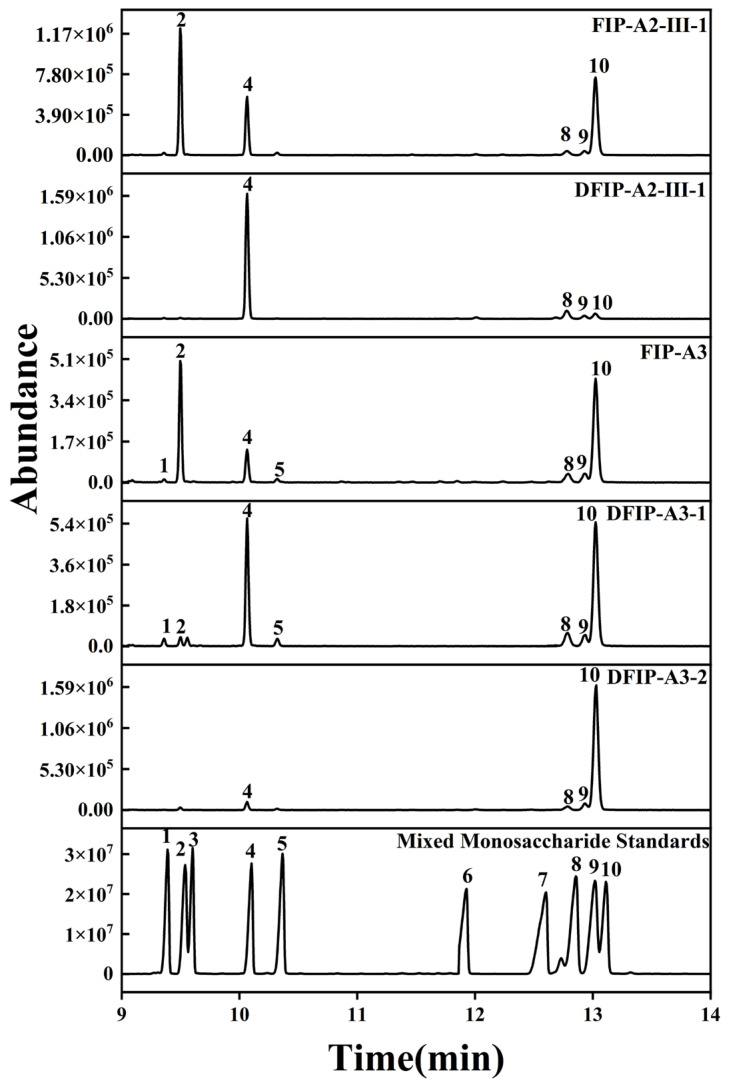
Monosaccharide compositions of FIP-A2-III-1, DFIP-A2-III-1, FIP-A3, DFIP-A3-1 and DFIP-A3-2. 1: Xyl, 2: Ara, 3: Rib, 4: Rha, 5: Fuc, 6: GalA, 7: GlcA, 8: Man, 9: Glc, 10: Gal.

To determine their monosaccharide composition, FIP-A2-III-1, FIP-A3, DFIP-A3-1, DFIP-A3-2, and DFIP-A2-III-1 were analyzed by gas chromatography-mass spectrometry (GC-MS). The resulting data are presented in Figure 4. According to the analytical results, FIP-A2-III-1 was found to be composed of Ara, Rha, Man, Glc, and Gal, with the molar ratio of 36.000:11.317:0.054:0.050:40.415. DFIP-A2-III-1 was found to be composed of Rha, Man, Glc, and Gal, with the molar ratio of 10.041:0.051:0.005:0.029. FIP-A3 was found to be composed of Xyl, Ara, Rha, Fuc, Man, Glc, and Gal, with the molar ratio of 0.003:6.161:7.255:0.005:0.084:0.062:13.993, while DFIP-A3-1 was found to be composed of Xyl, Ara, Rha, Fuc, Man, Glc, and Gal, with the molar ratio of 0.029:0.041:10.98:0.035:0.214:0.120:22.442. DFIP-A3-2 was found to be composed of Rha, Man, Glc, and Gal, with the molar ratio of 0.042:0.012:0.033:19.507. FIP-A2-III-1, DFIP-A2-III-1, FIP-A3, DFIP-A3-1 and DFIP-A3-2 exhibited differences in monosaccharide content. A comparison of the monosaccharide compositions before and after degradation revealed that Xyl, Ara, and Fuc were highly susceptible to hydrolysis. Therefore, these results indicated that three polysaccharides were present in the side chains and that none contained glucuronic acid or galacturonic acid. Consequently, FIP-A2-III-1, DFIP-A2-III-1, FIP-A3, DFIP-A3-1 and DFIP-A3-2 were determined to be neutral polysaccharides with Rha and Gal comprising the polysaccharide backbone.

The previous literature has demonstrated that FIP-II, another neutral polysaccharide from Isatidis Folium, exhibits an essentially identical monosaccharide composition, with similarly high proportions of Gal, Ara, and Rha [8]. Previous studies on polysaccharides extracted from the leaves of *Isatis indigotica* Fort. indicated that the neutral fraction WFIP-N contained high proportions of Gal and Rha. These findings provided data support for the polysaccharides obtained in the present study [36]. Based on numerous studies on the immunological activity of polysaccharides, it could be inferred that a high proportion of Gal and Rha is a crucial structural feature for exerting immunological activity [37].

### 2.6. Methylation Analysis

As shown in Table 2, T-Gal*p*, 1,6-Gal*p*, 1,3-Gal*p*, and 1,3,6-Gal*p* residues were simultaneously detected in FIP-A3, DFIP-A3-1, and DFIP-A3-2. Furthermore, FIP-A3 was found to contain substantial amounts of 1,4-Gal*p*, 1,3-Rha*p*, 1,2,4-Rha*p*, and 1,5-Ara*f* residues, along with minor quantities of T-Ara*f*. In DFIP-A3-1, 1,4-Gal*p* and 1,2,4-Rha*p* residues were also present. These findings further confirmed the structural differences among FIP-A3, DFIP-A3-1, and DFIP-A3-2. Unlike the findings from the monosaccharide composition analysis, the low-concentration monosaccharides detected in the composition data were not reflected in the methylation results, a phenomenon closely related to their relative abundance. FIP-A3, DFIP-A3-1, and DFIP-A3-2 were found to share similar backbone structures. From the aerial parts of *Astragalus membranaceus*, thirteen immunologically active polysaccharides were extracted and isolated. Among them, within an arabinogalactan possessing a backbone of β-1,3,6-Gal, it was observed that the degradation of either β-1,3-Gal or β-1,6-Gal residues significantly reduced its immunological activity [38]. A novel galactose-rich GLP-1b was successfully isolated and identified from *Ganoderma lucidum*. Its backbone was found to be primarily composed of 1,6-Gal and various terminal residues. It was further demonstrated that GLP-1b exerted its immunomodulatory effects through binding to the TLR4 [39]. Consequently, the precise configuration of glycosidic linkages within DFIP-A3-1 might be correlated with its immunomodulatory functions, given its nature as a neutral polysaccharide.

### 2.7. NMR Analysis

**Figure 5:**
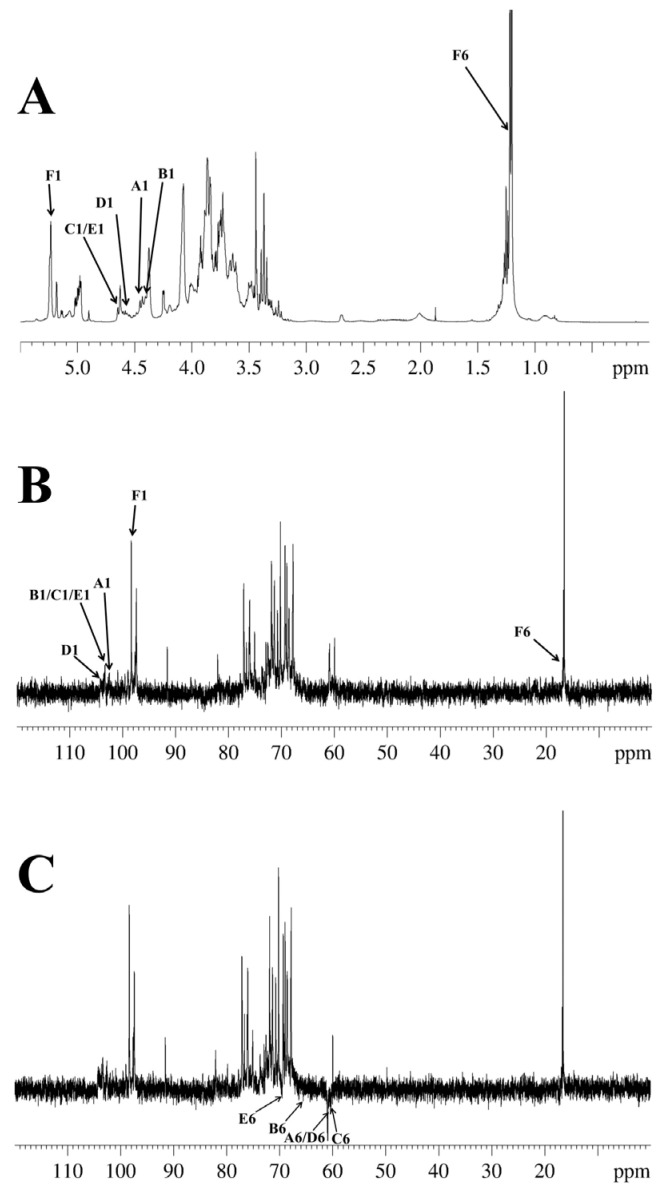
1D-NMR of DFIP-A3-1. (**A**) ^1^H NMR spectra (400 MHz). (**B**) ^13^C NMR spectra (100 MHz). (**C**) DEPT-135 spectra (100 MHz).

In agreement with earlier reports [40,41,42,43,44], six types of glycosidic linkages were determined in DFIP-A3-1 on the basis of anomeric proton resonances observed at *δ* 4.49, 4.45, 4.68, 4.58, 4.69 and 5.21. Signals for Xyl, Ara, Fuc, Man, and Glc residues were absent due to their low abundance (Figure 5A). The combined methylation and previously reported data suggested the presence of T-Gal*p* (A), 1,6-Gal*p* (B), 1,3-Gal*p* (C), 1,4-Gal*p* (D), 1,3,6-Gal*p* (E), and 1,2,4-Rha*p* (F). Complete ^1^H and ^13^C signal assignments were achieved via 1D/2D NMR (^1^H, ^13^C, DEPT-135, ^1^H−^1^H COSY, HSQC, HMBC and NOESY).

**Figure 6:**
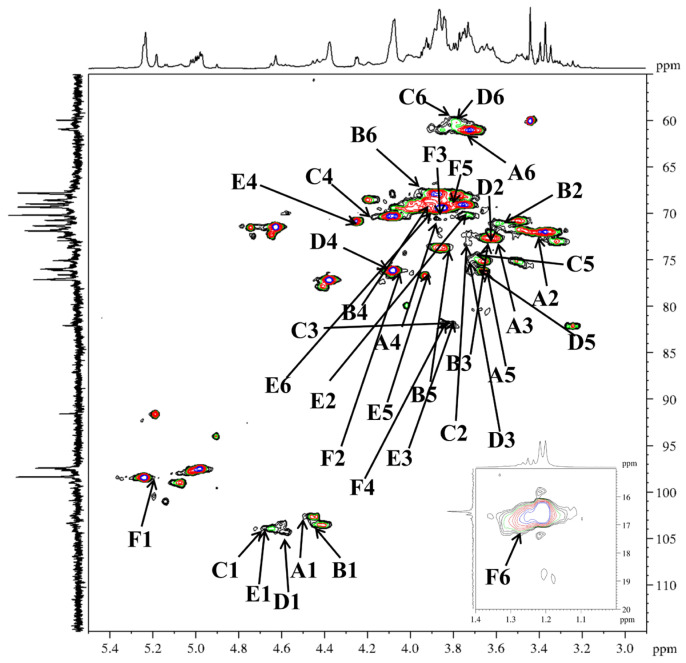
HSQC spectra of DFIP-A3-1.

**Figure 7:**
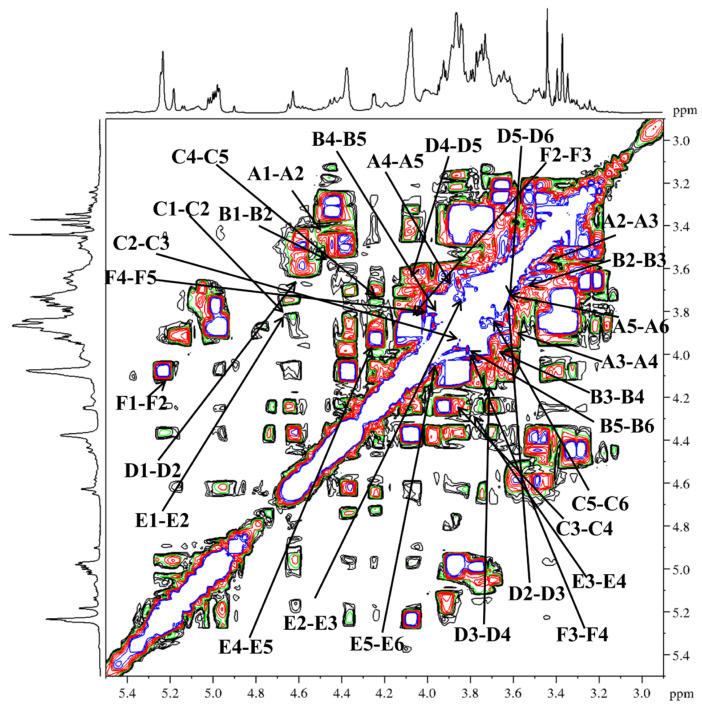
^1^H−^1^H COSY spectra of DFIP-A3-1.

Based on the HSQC spectrum (Figure 6), the cross-peaks at δ 4.49/102.4, 4.45/103.7, 4.68/103.9, 4.58/104.4, 4.69/103.8 and 5.21/98.7 were attributed to the anomeric correlations of residues A-F, which correspond to T-Gal*p*, 1,6-Gal*p*, 1,3-Gal*p*, 1,4-Gal*p*, 1,3,6-Gal*p* and 1,2,4-Rha*p* residues. Using the ^13^C NMR spectrum (Figure 5B), the chemical shifts in the corresponding anomeric carbons were subsequently determined. Key carbon signals for residues of −CH_2_ were further assigned using the DEPT-135 spectrum (Figure 5C). For instance, the inverted signals at *δ* 61.5, 67.4, 61.1, 61.4, and 69.8 were attributed to the C-6 atoms of residues A, B, C, D, and E. Notably, the anomeric proton chemical shifts in residues A–E were all below *δ* 4.95, indicating a β-configuration for these pyranose residues [45]. The anomeric proton chemical shift in residue F was observed at a relatively low field (*δ* 5.21), indicating an α-configuration [46]. Sequential assignment of the chemical shifts for H2–H6 and C2–C6 was accomplished by beginning with the anomeric proton signal of each residue, utilizing the scalar couplings between adjacent protons observed in the ^1^H−^1^H COSY spectrum (Figure 7) and the one-bond H−C connectivities derived from the HSQC spectrum. Beginning with the anomeric proton of residue T-β-Gal*p* (A), a complete chain of spin-spin couplings was traced through the ^1^H−^1^H COSY spectrum. This sequential assignment established the chemical shifts for the coupled proton pairs H-1/H-2 (*δ* 3.45), H-2/H-3 (*δ* 3.59), H-3/H-4 (*δ* 3.89), H-4/H-5 (*δ* 3.65), and H-5/H-6a, H-6b (*δ* 3.65/3.72,3.64). The direct H−C correlations observed in the HSQC spectrum were used to assign the corresponding carbon chemical shifts, which were assigned to C-2 (*δ* 72.9), C-3 (*δ* 73.0), C-4 (*δ* 69.7), C-5 (*δ* 75.9), and C-6 (*δ* 61.5). By applying this well-established methodology, the full set of H and C chemical shifts for residues B through E was systematically assigned; these data are summarized in Table 3. In the ^1^H NMR spectrum, the H-6 protons of the 1,2,4-α-Rha*p* (F) residue were observed at *δ* 1.27, and the corresponding C-6 signal was assigned at *δ* 17.3 based on the HSQC correlation.

**Figure 8:**
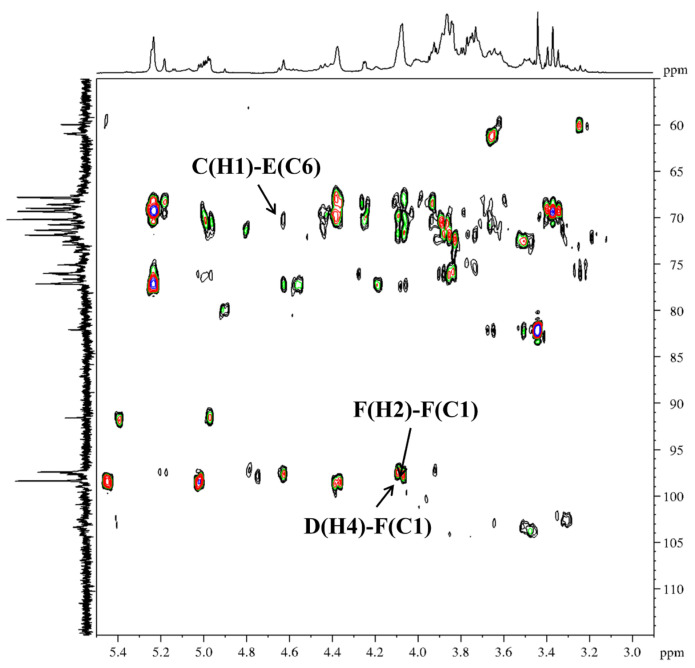
HMBC spectra of DFIP-A3-1.

**Figure 9:**
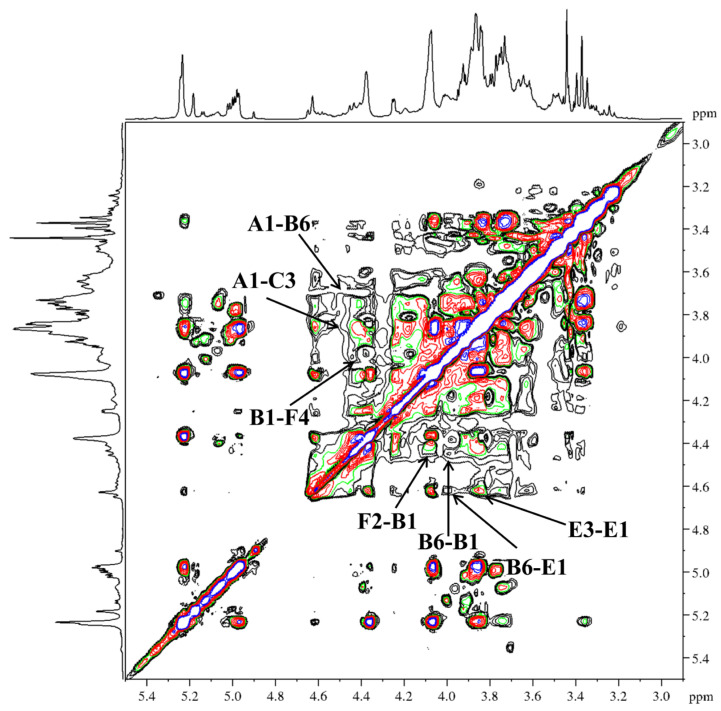
NOESY spectra of DFIP-A3-1.

**Figure 10:**
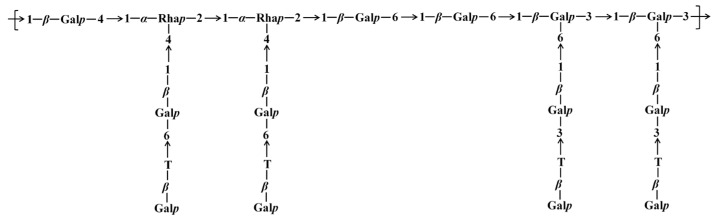
Putative structure of DFIP-A3-1.

Further examination of the HMBC (Figure 8) (*δ*_H/C_) and NOESY (Figure 9) (*δ*_H/H_) correlation signals revealed that the peak correlating 1,4-β-Gal*p* (D) H-4 with 1,2,4-α-Rha*p* (F) C-1 was located at *δ* 4.10/98.7. The correlation peak between H-2 and C-1 of 1,2,4-α-Rha*p* (F) was observed at *δ* 4.09/98.7. The correlation peak between H-2 of 1,2,4-α-Rha*p* (F) and H-1 of 1,6-β-Gal*p* (B) was observed at *δ* 4.09/4.45. The Ha-6 and H-1 correlation peak of 1,6-β-Gal*p* (B) was observed at *δ* 3.96/4.45. The correlation peak between Ha-6 of 1,6-β-Gal*p* (B) and H-1 of 1,3,6-β-Gal*p* (E) was observed at *δ* 3.96/4.69. The correlation peak between H-3 and H-1 of 1,3,6-β-Gal*p* (E) was located at *δ* 3.85/4.69. It was indicated by the combined evidence that the backbone structure of DFIP-A3-1 was composed of →1)-β-Galp-(4→1,4)-α-Rhap-(2→1,4)-α-Rhap-(2→1)-β-Galp-(6→1)-β-Galp-(6→1,6)-β-Galp-(3→1,6)-β-Galp-(3→, with branches at the O-4 position of 1,2,4-α-Rha*p* and the O-6 position of 1,3,6-β-Gal*p*. A cross-peak between H-1 of T-β-Gal*p* (A) and Hb-6 of 1,6-β-Gal*p* (B) was observed at *δ* 4.49/3.67. A cross-peak between H-1 of 1,6-β-Gal*p* (B) and H-4 of 1,2,4-α-Rha*p* (F) was observed at *δ* 4.45/4.01. A correlation between H-1 of T-β-Gal*p* (A) and H-3 of 1,3-β-Gal*p* (C) was detected at *δ* 4.49/3.85. Additionally, a cross-peak between H-1 of 1,3-β-Gal*p* (C) and C-6 of 1,3,6-β-Gal*p* (E) was observed at *δ* 4.68/69.8. Collectively, these correlations reveal the branched structure of DFIP-A3-1. The putative repeating unit structure of DFIP-A3-1 is shown in Figure 10. Previous studies on plant-derived polysaccharides have also reported similar backbone and branching structures. For instance, a neutral polysaccharide (DPSW-A) was isolated from *Taraxacum mongolicum* Hand.-Mazz., and it was hydrolyzed with TFA to yield DPSW-A-on and DPSW-A-out. The backbone of DPSW-A-on was found to consist of →6)-β-Gal*p*-(1→, →2,4)-α-Rha*p*-(1→, and →3,6)-β-Gal*p*-(1→ residues, with branches attached at the O-4 position of 1,2,4-α-Rha*p* and the O-6 position of 1,3,6-β-Gal*p* residue. The O-6 position of the →3,6)-β-Gal*p*-(1→ residue served as the attachment site for a T-β-Gal*p*-(1→3)-β-Gal*p*-(1→ side chain. In a parallel manner, the dominant components of the hydrolyzed fragments were identified as T-α-Ara*f*-(1→ and →5)-α-Ara*f*-(1→ residues [47]. A polysaccharide, designated as REPI, was isolated from radish leaves. Its backbone was found to be composed of repeating units of →2)-Rha*p*-(1→4)-Gal*p*A-(1→, along with three side chains. Among these, →4)-β-Gal*p*-(1→ was attached at the O-4 position of →2,4)-α-Rha*p*-(1→ residue, and an arabino-β-3,6-galactan was also linked to the O-4 position of the same →2,4)-α-Rha*p*-(1→ residue [48].

### 2.8. Congo Red Assay Analysis

**Figure 11:**
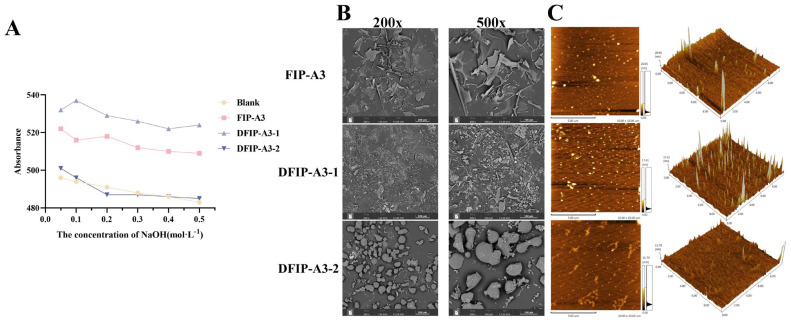
Congo red staining and surface morphology analysis of FIP-A3, DFIP-A3-1 and DFIP-A3-2. (**A**) Congo red results for FIP-A3, DFIP-A3-1 and DFIP-A3-2. (**B**) 200× and 500× SEM results for FIP-A3, DFIP-A3-1 and DFIP-A3-2. (**C**) AFM results for FIP-A3, DFIP-A3-1 and DFIP-A3-2.

As shown in Figure 11A, the polysaccharides derived from the three-component fraction formed corresponding complexes with Congo red. As the NaOH concentration was increased, FIP-A3 and DFIP-A3-1 exhibited an initial rise followed by a sharp decline in their λ_max_ values, suggesting the potential presence of a triple-helix conformation. In contrast, DFIP-A3-2 did not exhibit this initial increase followed by a sharp decrease, suggesting it likely lacks a triple-helix structure.

The triple-helical conformation of the polysaccharide is stabilized mainly by intermolecular hydrogen bonds and interactions with solvent molecules [49]. A comparative study demonstrated that, relative to FVPU1, FVPU2 possessed a more stable triple-helix conformation and displayed superior anti-inflammatory properties, primarily by attenuating the aberrant upregulation of inflammatory mediators, including NO, IL-1β, IL-6, and TNF-α [50]. *Dendrobium officinale* was the source of DLP-1 and DLP-2. Interestingly, both were found to have a triple-helix structure and had superior immunomodulatory bioactivity, promoting RAW 264.7 cell proliferation, phagocytic ability, and immune factor synthesis [51]. These results suggested that the triple-helical structure was linked to its bioactivity.

### 2.9. SEM Analysis

As shown in Figure 11B, distinct differences in the morphological features of FIP-A3, DFIP-A3-1, and DFIP-A3-2 were revealed by scanning electron microscopy (SEM) under 200× and 500× magnification. Upon examination, FIP-A3 displayed large, plate-shaped morphologies characterized by a disordered arrangement, subtle surface undulations, and comparatively even margins. DFIP-A3-1 was observed to have a rough surface with wrinkles and uneven edges, while DFIP-A3-2 was observed to display a rough surface with fissures, presenting a honeycomb-like structure.

### 2.10. AFM Analysis

As shown in Figure 11C, atomic force microscopy (AFM) analysis of the polysaccharide molecular morphology revealed that all three polysaccharide components—FIP-A3, DFIP-A3-1, and DFIP-A3-2—exhibited relatively uniform granular and network-like structures in 2D planar images. The 3D images revealed that the polysaccharide surfaces were densely covered with sharp peak-like structures, with peak heights of 20.65 nm, 17.41 nm, and 11.79 nm. Relevant studies indicated that the height of single chains in polysaccharides typically ranges from 0.1 to 1.0 nm [52]. Therefore, it was inferred that FIP-A3, DFIP-A3-1, and DFIP-A3-2 all possessed long branched chains and stacked multi-chain structures, along with good molecular aggregation properties.

### 2.11. Immunomodulatory Activity In Vitro of FIP

#### 2.11.1. RAW 264.7 Macrophage Proliferation

The in vitro activities of FIP-A, FIP-B, FIP-C, FIP-D, and FIP-E, which were obtained by membrane separation, were carefully determined. As shown in Figure S3, FIP-D exhibited the strongest immune-enhancing effect, followed by FIP-A. Due to the extremely low yield of FIP-D after membrane separation, FIP-A was selected for further processing.

**Figure 12:**
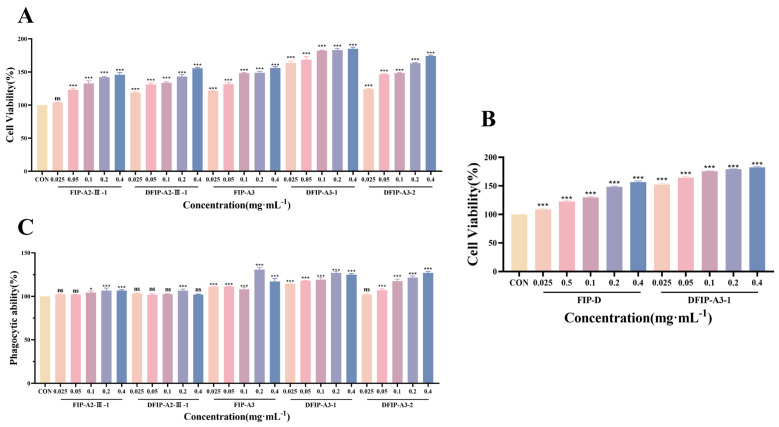
Screening results of immunomodulatory activity of polysaccharide fractions on RAW 264.7 cells. (**A**): Screening of immunological activity of FIP-A2-III-1, DFIP-A2-III-1, FIP-A3, DFIP-A3-1 and DFIP-A3-2 on RAW 264.7 cells. ($\bar{x}$ ± s; *n* = 3) *** *p* < 0.001 vs. the control and ns indicates not statistically significant. (**B**) Screening of immunological activity of FIP-D and DFIP-A3-1 on RAW 264.7 cells. ($\bar{x}$ ± s; *n* = 3) *** *p* < 0.001 vs. the control. (**C**) Effects of FIP-A2-III-1, DFIP-A2-III-1, FIP-A3, DFIP-A3-1 and DFIP-A3-2 on phagocytic activity in RAW 264.7 cells. ($\bar{x}$ ± s; *n* = 3) * *p* < 0.05, *** *p* < 0.001 vs. the control and ns indicates not statistically significant.

As shown in Figure 12A, the FIP-A2-III-1, DFIP-A2-III-1, FIP-A3, DFIP-A3-1 and DFIP-A3-2 were subjected to CCK-8 assays before and after degradation. The results revealed that DFIP-A3-1 exhibited significant immunostimulatory activity (*p* < 0.001). These data suggest that the immune-regulating potency of polysaccharides is tightly associated with their molecular weight [53,54,55,56]. Polysaccharides with higher molecular weights possess greater steric hindrance and lower solubility. Their active groups become encapsulated and cannot fully expose themselves for binding to receptors, leading to reduced bioavailability and consequently diminished immunological activity [57]. Lower-molecular-weight polysaccharides show stronger transmembrane penetration and an enhanced ability to trigger biological responses. However, an excessively low molecular weight may reduce spatial folding, compromising the maintenance of high-order structures. This results in short, irregular, highly flexible linear or globular conformations [58]. This structural feature likely explains why DFIP-A3-1 demonstrates optimal immunostimulatory effects.

Simultaneously, as shown in Figure 12B, the most active degraded polysaccharide, DFIP-A3-1, was compared with FIP-D in terms of activity. The results indicate that the degraded polysaccharide exhibited superior immune-enhancing effects (*p* <0.001). This study did not directly pursue the naturally occurring, extremely low-yield, highly active component FIP-D. Instead, it utilized the high-yield, high-molecular-weight component FIP-A as a precursor. The degraded polysaccharide DFIP-A3-1 was obtained by acid degradation and was found to exhibit significantly higher immunological activity than its precursor. This approach effectively overcomes the bottleneck of limited natural high-activity polysaccharide resources.

#### 2.11.2. Phagocytic Ability Assay

As shown in Figure 12C, phagocytosis is a hallmark of macrophage activation [59,60]. The impact of FIP-A2-III-1, DFIP-A2-III-1, FIP-A3, DFIP-A3-1, and DFIP-A3-2 on the phagocytic function of RAW264.7 cells was assessed in the present study using a neutral red phagocytosis assay. The results indicated that the polysaccharides from these fractions enhanced macrophage phagocytic rates in a concentration-dependent manner. This demonstrated that FIP-A2-III-1, DFIP-A2-III-1, FIP-A3, DFIP-A3-1, and DFIP-A3-2 effectively activated macrophages.

DFIP-A3-1 demonstrated strong phagocytic capacity overall (*p* < 0.001). Notably, the level of phagocytic activity recorded at 0.4 mg/mL did not differ significantly from that observed at 0.2 mg/mL, an outcome that aligns closely with the data obtained from the CCK-8 assay.

#### 2.11.3. Determination of NO, IL-6 and TNF-α

**Figure 13:**
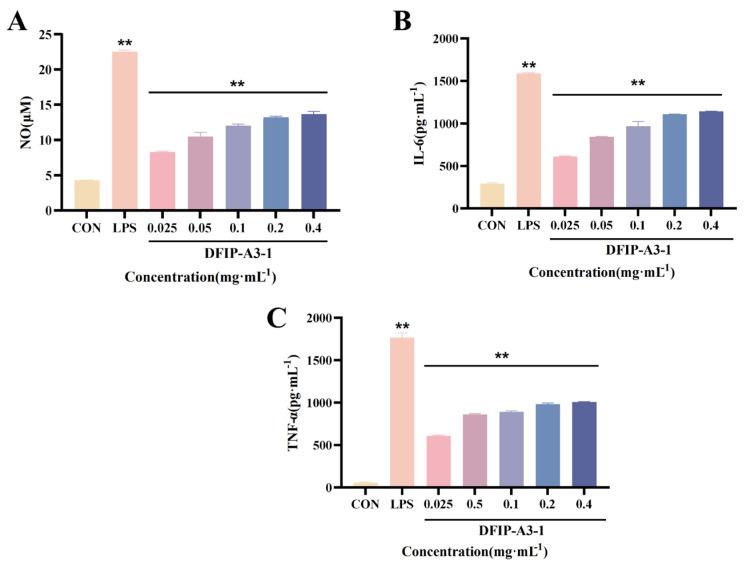
The effects of DFIP-A3-1 on the secretion of NO, IL-6 and TNF-α in RAW 264.7 cells. (**A**) NO. (**B**) IL-6. (**C**) TNF-α. ($\bar{x}$ ± s; *n* = 3) ** *p* < 0.01 vs. the control.

NO, IL-6, and TNF-α are key mediators produced upon macrophage activation, playing central roles in innate immunity and inflammatory responses to identify and eliminate pathogens, thereby providing essential protection for the body [61,62,63]. Following treatment, the levels of immunostimulatory cytokines released from RAW264.7 cells were quantified; the corresponding data are presented in Figure 13. Relatively low secretion of NO, IL-6, and TNF-α was detected in the control group. In contrast, the production of these cytokines was significantly increased by lipopolysaccharide (LPS) (*p* < 0.01). After 24 h of exposure to DFIP-A3-1, a significant concentration-dependent release of NO, IL-6, and TNF-α was detected (*p* < 0.01).

#### 2.11.4. NO, IL-6, and TNF-α mRNA Expression

**Figure 14:**
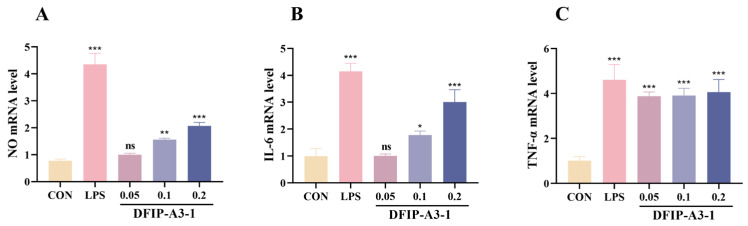
Expressions of NO, IL-6 and TNF-α mRNA in RAW 264.7 cells. (**A**) NO. (**B**) IL-6. (**C**) TNF-α. ($\bar{x}$ ± s; *n* = 3) * *p* < 0.05, ** *p* < 0.01, *** *p* < 0.001 vs. the control and ns indicates not statistically significant.

Furthermore, the mechanism by which DFIP-A3-1 stimulates macrophages to secrete immunostimulatory factors through the regulation of gene expression was investigated. After treatment with DFIP-A3-1, a significant upregulation in the expression of NO, IL-6, and TNF-α was observed, as depicted in Figure 14. These findings indicated that DFIP-A3-1 effectively induced immunomodulatory activity (*p* < 0.01).

#### 2.11.5. Mechanism of Action of DFIP-A3-1 in Activating the NF-κB Pathway

In this study, the activation of the nuclear factor kappaB (NF-κB) signaling pathway was evaluated to elucidate toll-like receptor 4 (TLR4) as the primary receptor for DFIP-A3-1 binding and to determine the mechanism by which DFIP-A3-1 induced macrophage activation. This process involves TLR4 binding to its corresponding ligand, activating downstream kinases (such as IRAKs and TRAF6), which ultimately activate the key kinase complex TAK1. Phosphorylated TAK1 activates the IKK complex, including IKKβ, which directly phosphorylates serine residues on the IκBα protein. Phosphorylated IκBα (p-IκBα) is recognized by the proteasome and rapidly degraded. The NF-κB (p65–p50) dimer is then released and translocated into the nucleus, where p65 undergoes phosphorylation, ultimately activating the NF-κB pathway [4,64,65].

**Figure 15:**
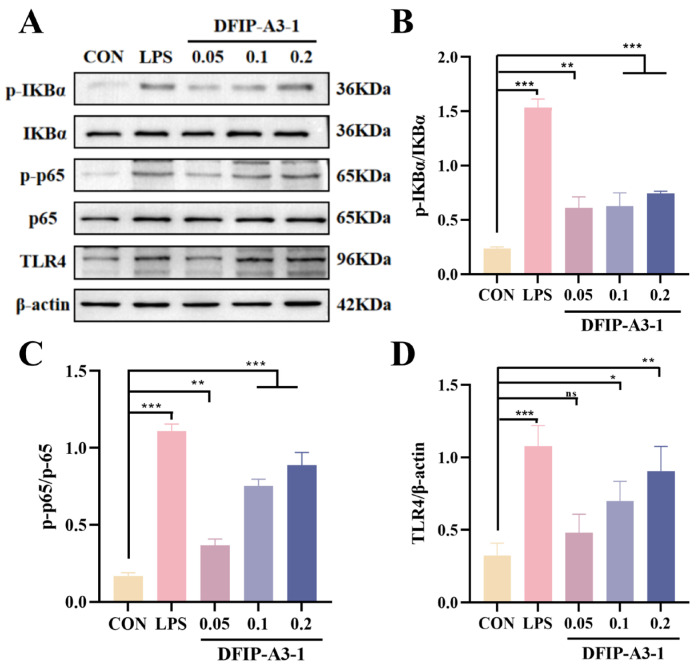
The p-IKKα, IKKα, p-p65, p65 and TLR4 protein expression levels in RAW264.7 cells. (**A**) The relative protein quantification of p-IκBα, p-p65, and TLR4. (**B**) p-IKKα and IKKα. (**C**) p-p65 and p65. (**D**) TLR4. ($\bar{x}$ ± s; *n* = 3), * *p* < 0.05, ** *p* < 0.01, *** *p* < 0.001 vs. the control and ns indicates not statistically significant.

**Figure 16:**
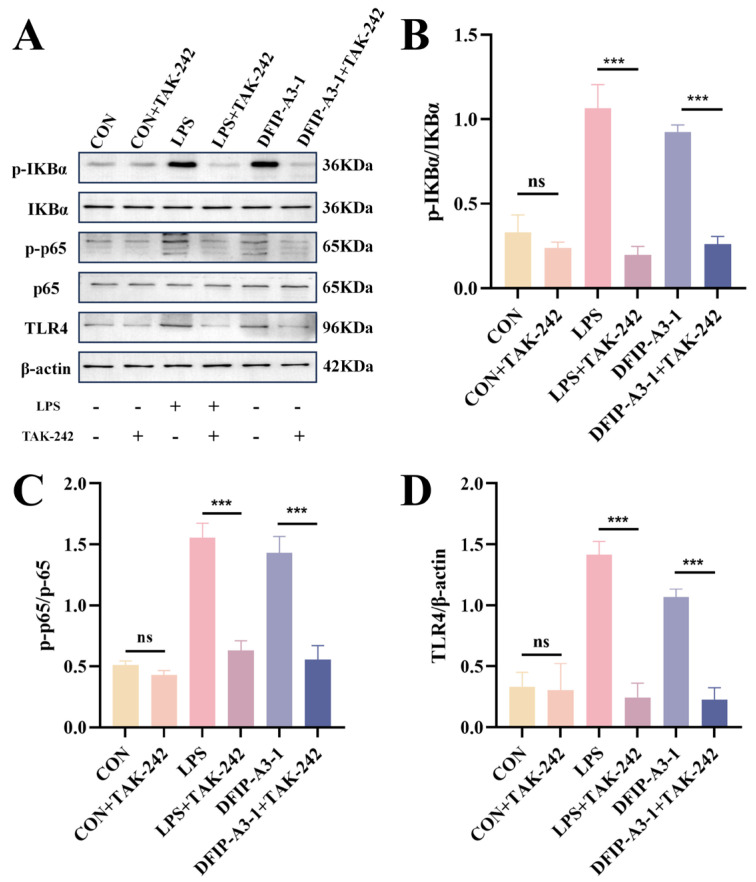
TAK242 inhibition downregulates the expression levels of p-IKKα, IKKα, p-p65, p65 and TLR4 proteins in RAW264.7 cells. (**A**) The relative protein quantification of p-IκBα, p-p65, and TLR4. (**B**) p-IKKα and IKKα. (**C**) p-p65 and P65. (**D**) TLR4. ($\bar{x}$ ± s; *n* = 3), *** *p* < 0.001 vs. the control and ns for not statistically significant.

**Figure 17:**
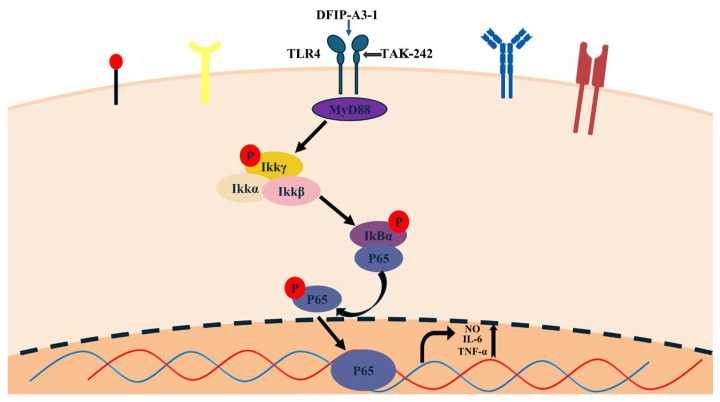
Schematic diagram of the immunomodulatory mechanism of DFIP-A3-1.

As shown in Figure 15, it was revealed by Western blot analysis that DFIP-A3-1 significantly enhanced the phosphorylation levels of IκBα and p65 (*p* < 0.01). This finding further suggested that TLR4 played a critical role in the DFIP-A3-1-mediated activation of RAW264.7 cells. To further verify this conclusion, the specific TLR4 inhibitor TAK-242 was added to the cells [66,67,68]. As shown in Figure 16, TAK-242 treatment significantly antagonized the DFIP-A3-1-induced upregulation of p-IκBα, p-p65, and TLR4 expression. To further clarify the immunomodulatory properties of DFIP-A3-1, a schematic diagram of its potential mechanism is provided in Figure 17.

Some limitations were still present in this experiment. For instance, the stereostructural elucidation of DFIP-A3-1 was not precisely achieved. Owing to the complexity of the molecular weight and structure of polysaccharides, as well as the variability of their stereoconfigurations, the spatial conformation was difficult to determine. Similarly, due to the complexity of the signaling pathways, only one NF-κB pathway was identified through literature review in this study, and the possibility of the involvement of other pathways could not be excluded. Subsequent in vivo pharmacological experiments could be conducted to further verify the mechanism underlying its immunomodulatory activity. Meanwhile, in the context of scaled-up production and application, the experimental procedures could be refined, and effective translation into industrialization could be achieved.

## 3. Materials and Methods

### 3.1. Materials and Reagents

Isatidis Folium plants were cultivated in Bajingzi Township, Datong District, Daqing City, Heilongjiang Province. The RAW264.7 cell line was purchased from Haixing Biotechnology (Suzhou, China). All analytical reagents and standard monosaccharides utilized in this experiment were commercially available. The sources and specifications of experimental reagents were consistent with our previous published research [69].

### 3.2. Preparation, Isolation and Purification

The leaves of *Isatis indigotica* Fort. were defatted with 70% ethanol, and the residue was subsequently extracted with hot water. The resulting aqueous extract was concentrated and then lyophilized. AB-8 macroporous adsorbent resin was used for further decolorization and deproteinization, and the crude polysaccharides were obtained and designated as FIP. FIP (140 g) was dissolved and then sequentially fractionated using 100, 50, 20, and 10 kDa ultrafiltration membranes (BONA-GM-20, BONA GROUP, Jinan, China). The resulting fractions were designated as FIP-A to FIP-E. FIP-A was further subjected to ethanol fractional precipitation by adjusting the ethanol concentration to 40%, 60% and 80% respectively, and the resulting fractions were named FIP-A1~3. FIP-A2 was purified on a DEAE-650M cellulose column (5 cm × 60 cm; purchased from Tosoh Corporation, Tokyo, Japan) eluted with a linear gradient of 0–0.5 M NaCl solution, yielding sub-fractions designated as FIP-A2-I to FIP-A2-V. FIP-A2-III was further purified using Sephadex G-100 (Cytiva, Marlborough, MA, USA) gel column chromatography to obtain FIP-A2-III-1.

### 3.3. Acid Hydrolysis

FIP-A2-III-1 and FIP-A3 (100 mg) were dissolved in 0.5 M TFA (4 mL) and hydrolyzed in an oven at 80 °C for 2 h. The resulting products were then co-evaporated with methanol to eliminate residual acid. The samples were dissolved in ultrapure water (10 mL) and freeze-dried. Prior to use, each degraded polysaccharide was quantified and subsequently passed through a 0.8 μm membrane filter. Fractionation and isolation were subsequently achieved via column chromatography on a Toyopearl HW-55F column (Tosoh, Tokyo, Japan). The Waters 515-2414 HPLC system (Milford, MA, USA) was employed for the isolation and preparation of the degraded polysaccharides. The eluent was collected at a flow rate of 1.0 mL/min using an automatic fraction collector at intervals of 5 min per tube over a total run time of 600 min, yielding 120 fractions. After combining appropriate tubes and freeze-drying, three fractions were obtained and designated as DFIP-A2-III-1, DFIP-A3-1, and DFIP-A3-2, respectively.

### 3.4. Homogeneity and Molecular Weight

To evaluate homogeneity, an adapted version of a method described previously was employed [70]. Analysis was performed on an HPGPC system equipped with an Ultrahydrogel^TM^ Linear column (7.8 mm × 300 mm, Tosoh, Tokyo, Japan) connected to an HPLC-evaporative light-scattering detector (ELSD, Agilent 1260II HPLC, c.01.08.210, Agilent Technologies, Santa Clara, CA, USA).

According to a previously reported method [71], the molecular weights (*M*_w_) of FIP-A2-III-1, DFIP-A2-III-1, FIP-A3, DFIP-A3-1, and DFIP-A3-2 were determined using HPSEC-MALLS-RID.

### 3.5. FT-IR Spectrum

For FT-IR spectroscopic analysis, each polysaccharide sample (3 mg) was thoroughly mixed with 200 mg of dried KBr. The mixture was subsequently compressed into a transparent disc. Spectral measurements were then performed over the range of 400–4000 cm^−1^ using an FTIR spectrophotometer (VERTEX-70, FTR 5.4.9, Bruker, Rheinstetten, Germany).

### 3.6. Monosaccharide Compositions

The monosaccharide composition of the polysaccharides was established via a method adapted from previously reported protocols [72]. Briefly, the polysaccharide samples were kindly hydrolyzed with 2 M TFA at 110 °C for 4 h, followed by derivatization together with a mixed standard of monosaccharides. The temperature program for TMSA derivatization was set as follows: an initial hold at 180 °C for 0 min, followed by ramping at 4 °C/min to 190 °C, then at 1 °C/min to 200 °C, and subsequently at 2 °C/min to 230 °C and further to 300 °C, with a final hold of 5 min.

### 3.7. Methylations

According to a previously reported method [73]. The lyophilized samples—FIP-A2-III-1, DFIP-A2-III-1, FIP-A3, DFIP-A3-1, and DFIP-A3-2 (2 mg)—were subjected to complete methylation. This was followed by hydrolysis, reduction, acetylation, and subsequent analysis employing a GC–MS system (Agilent 7890A-5975C, version 10.0, Agilent Technologies, Santa Clara, CA, USA) outfitted with a DB-5 silica capillary column (60 m × 0.25 mm × 0.25 µm). The temperature program employed for GC–MS analysis consisted of the following steps: an initial hold at 80 °C for 1 min, followed by a ramp to 200 °C at 5 °C/min, then to 220 °C at 2 °C/min, and finally to 270 °C at 10 °C/min, where the temperature was maintained for an additional 5 min.

### 3.8. 1D and 2D NMR

For NMR spectroscopic analysis, DFIP-A3-1 was solubilized in deuterium oxide (D_2_O). Subsequently, both one-dimensional and two-dimensional NMR spectra were acquired using a Bruker AM-400 spectrometer (operating at 400 MHz, version 11.1, Bruker, Fällanden, Switzerland).

### 3.9. Congo Red Test

Accurately weighed polysaccharide samples were dissolved in deionized water. Subsequently, each sample solution was combined with Congo red reagent (100 μM), and the mixture was titrated with 1 M NaOH to obtain final concentrations of NaOH of 0.05, 0.1, 0.2, 0.3, 0.4, and 0.5 M. After standing at room temperature for 10 min, the absorbance spectra of all mixtures were recorded over the 190–700 nm range with a UV-Vis spectrophotometer. The λ_max_ and the associated absorbance for each sample were then obtained at the various NaOH concentrations tested. A binding curve was constructed by plotting the maximum absorption wavelength (nm) against the NaOH concentration (M).

### 3.10. Scanning Electron Microscopy

SEM analysis was employed to examine the surface features of FIP-A3, DFIP-A3-1, and DFIP-A3-2 (JSM-7800F, JEOL Ltd., Tokyo, Japan). After drying, the specimens were mounted on a conductive substrate and sputter-coated with gold using a vacuum deposition system. Observations were carried out under high vacuum conditions at an appropriate accelerating voltage.

### 3.11. Atomic Force Microscopy

A solution of FIP-A3, DFIP-A3-1, and DFIP-A3-2 was prepared at 5 µg/mL in deionized water and subsequently passed through a 0.22 µm cellulose filter. An aliquot of this filtrate was then deposited onto a freshly cleaved mica substrate and allowed to dry at ambient temperature. Morphological observations of the samples were conducted by AFM using an SPM-9700 instrument (SPM-9700, version 2.61, Shimadzu, Kyoto, Japan). The imaging was carried out in tapping mode, covering a region of 1.000 × 1.000 µm at a scan rate of 1.00 Hz.

### 3.12. Immunoregulatory Activity Test

#### 3.12.1. Cell Culture and Treatment

RAW 264.7 cells represent a murine monocyte/macrophage lineage characterized by adherent growth and a rounded morphology. The culture environment consisted of DMEM medium containing 12% FBS and 0.1% penicillin-streptomycin, with cells maintained at 37 °C in a humidified incubator supplied with 5% CO_2_. Following 24 h of culture, cellular proliferation was monitored, and the medium was subsequently refreshed as needed.

#### 3.12.2. Cell Viability Assay

For the culture procedure, RAW264.7 cells were plated in 96-well microplates at an initial density of 1 × 10^5^ cells per well. Following a 24 h adhesion period, the adherent cells were incubated for an additional 24 h with varying concentrations of FIP-A, FIP-B, FIP-C, FIP-D, and FIP-E. These concentrations spanned a range of 0.025, 0.05, 0.1, 0.2, and 0.4 mg/mL. Thereafter, a 10% CCK-8 solution was introduced, and the incubation was extended for an additional 2 h. The absorbance at 450 nm, reflecting cell viability, was recorded with a microplate reader (SpectraMax ABS Plus, version 7.1.0; Molecular Devices, San Jose, CA, USA).

The same method was employed in the subsequent in vitro immunostimulatory activity screening of FIP-A2-III-1, DFIP-A2-III-1, FIP-A3, DFIP-A3-1, and DFIP-A3-2. The optimal fraction, DFIP-A3-1, identified from these results, was then subjected to a further in vitro immunostimulatory activity assay alongside the previously evaluated FIP-D fraction, following the same procedure.

#### 3.12.3. Phagocytosis Assay

The phagocytic activity of the cells was assessed using a neutral red assay kit. Cells were treated with varying concentrations of FIP-A2-III-1, DFIP-A2-III-1, FIP-A3, DFIP-A3-1, and DFIP-A3-2 for 24 h, with culture medium alone serving as the blank control. Subsequently, neutral red solution was added to the cells, followed by incubation for 2 h. After washing with PBS, cell lysis buffer (composed of 1% acetic acid and 50% ethanol, 1:1, *v*/*v*) was added. The absorbance was then measured at 540 nm.

#### 3.12.4. Cytokine Detection

The levels of NO, IL-6 and TNF-α in the cell culture supernatants were determined using the Griess assay (Beyotime, Shanghai, China) and ELISA kits (Jiangsu Yutong Biotechnology Co., Ltd., Yancheng, China), respectively, strictly following the manufacturers’ instructions. Following drug stimulation, the cell culture supernatants were collected, and their absorbance was measured at the specified wavelengths using a microplate reader. All experimental procedures were performed in accordance with previously reported methods [74].

#### 3.12.5. qRT-PCR Analysis

RNA was extracted following the supplier’s instructions, and reverse transcription was carried out according to the manufacturer’s protocol for the kit. Quantitative real-time PCR (qRT-PCR) was employed to assess mRNA expression levels. The reaction system and calculation method were kept consistent with those employed in our previous study [69] (Table 4).

#### 3.12.6. Western Blotting

In the present investigation, Western blotting was carried out [75]. Radioimmunoprecipitation assay buffer (RIPA buffer) was used to extract total proteins from RAW264.7 cells. After fractionation by sodium dodecyl-sulfate polyacrylamide gel electrophoresis (SDS-PAGE), the resolved proteins were electrotransferred onto polyvinylidene fluoride (PVDF) membranes. These membranes were subsequently probed overnight at 4 °C with primary antibodies directed against IκBα, p-IkBα, p65, p-p65 and TLR4. The membrane was washed with tris-buffered saline with Tween-20 (TBST) buffer containing Tween-20, and the blot was imaged using an Odyssey CLx gel imaging system (Tanon-4200, 2.6.0.0, Tanon, Shanghai, China).

To determine whether TLR4 is required for the activation of the NF-κB pathway in RAW264.7 cells, experiments were performed according to a modified protocol based on established methods [76]. Cells were pre-incubated with 20 μM TAK-242, a selective TLR4 antagonist, for 6 h. Following removal of the culture supernatant, the cells were subsequently challenged with LPS and DFIP-A3-1 for an additional 24 h. Protein isolation was carried out following the same procedure described above for subsequent experimental analysis.

### 3.13. Statistical Analysis

To analyze the data, one-way analysis of variance (ANOVA) followed by multiple comparison test was performed. All values were expressed as the mean ± standard error of the mean ($\bar{x}$ ± s). * *p* < 0.05, ** *p* < 0.01 and *** *p* < 0.001 were considered statistically significant, and ns for not statistically significant. Graphical data were generated and processed using GraphPad Prism 8.0 (GraphPad Software, Boston, MA, USA) and Origin 2022.

## 4. Conclusions

In conclusion, two homogeneous polysaccharides, designated as FIP-A2-III-1 and FIP-A3, were successfully extracted and purified from the leaves of *Isatis indigotica* Fort. Subsequent degradation of these two polysaccharides yielded three degraded polysaccharides: DFIP-A2-III-1, DFIP-A3-1, and DFIP-A3-2. Chemical and spectroscopic analyses were performed on FIP-A2-III-1, DFIP-A2-III-1, FIP-A3, DFIP-A3-1, and DFIP-A3-2, and their primary structures were elucidated. As a result, they were determined to be neutral polysaccharides with molecular weights and monosaccharide compositions. Notably, DFIP-A2-III-1, DFIP-A3-1, and DFIP-A3-2 exhibited varying degrees of reduction in both molecular weight and monosaccharide species compared to their parent polysaccharides.

Furthermore, DFIP-A3-1 demonstrated potent immunostimulatory activity in preliminary screenings based on in vitro immune and phagocytic assays. It was identified as a low-molecular-weight rhamnogalactan, with glycosidic linkages comprising T-Gal*p*, 1,6-Gal*p*, 1,3-Gal*p*, 1,4-Gal*p*, 1,3,6-Gal*p*, and 1,2,4-Rha*p*. NMR analysis revealed that the main chain structure was determined to be →1)-β-Galp-(4→1,4)-α-Rhap-(2→1,4)-α-Rhap-(2→1)-β-Galp-(6→1)-β-Galp-(6→1,6)-β-Galp-(3→1,6)-β-Galp-(3→, with branching occurring at the O-4 and O-6 positions of 1,2,4-β-Rhap and 1,3,6-β-Galp, respectively. The side chains were identified as T-β-Galp-(1→6)-β-Galp-(1→, and T-β-Galp-(1→3)-β-Galp-(1→. The in vitro immunostimulatory effects of DFIP-A3-1 were systematically evaluated. Within a defined concentration range, DFIP-A3-1 significantly enhanced the release of key immunomodulatory cytokines. Moreover, marked upregulation of the mRNA expression of NO, IL-6, and TNF-α was observed. Additionally, the levels of phosphorylated IκBα, phosphorylated p65, and TLR4 were markedly upregulated. This upregulation was effectively suppressed by treatment with TAK-242, thereby validating the essential involvement of the TLR4 receptor in DFIP-A3-1-induced macrophage activation and subsequent immune modulation.

Subsequent investigations should aim to evaluate the in vivo immunomodulatory efficacy of DFIP-A3-1. The present work establishes a rigorous scientific foundation for understanding the structure-activity relationship of this polysaccharide and underscores its promise as a candidate for natural immunotherapeutic applications.

## Figures and Tables

**Table 1:** Molecular weight data for FIP-A2-III-1, DFIP-A2-III-1, FIP-A3, DFIP-A3-1 and DFIP-A3-2.

(g/mol)	FIP-A2-III-1	DFIP-A2-III-1	FIP-A3	DFIP-A3-1	DFIP-A3-2
*M*_n_	1.98 × 10^5^	3.52 × 10^3^	9.03 × 10^4^	5.64 × 10^3^	1.75 × 10^3^
*M*_p_	2.60 × 10^5^	3.54 × 10^3^	8.41 × 10^4^	5.72 × 10^3^	9.96 × 10^3^
*M*_w_	2.29 × 10^5^	5.11 × 10^3^	9.85 × 10^4^	8.68 × 10^3^	2.24 × 10^3^
*M*_z_	2.64 × 10^5^	1.04 × 10^4^	1.15 × 10^5^	1.54 × 10^4^	3.18 × 10^3^
*M*_w_/*M*_n_	1.16	1.43	1.09	1.54	1.28

**Table 2:** The glycosidic linkage types and molar percentages of FIP-A3, DFIP-A3-1 and DFIP-A3-2 based on the methylation and GC–MS analyses.

PMAA	Type of Glycosidic Bond	Mass Fragments (*m*/*z*)	Mol (%)		
			FIP-A3	DFIP-A3-1	DFIP-A3-2
2,3,4,6-Me_3_-Gal*p*	T-Gal*p*	59, 71, 87, 102, 118, 129, 145, 174, 205	0.38	1.14	1.54
2,3,4-Me_3_-Gal*p*	1,6-Gal*p*	59, 71, 99, 118, 129, 143, 159, 173, 189	2.32	2.20	8.80
2,4,6-Me_3_-Gal*p*	1,3-Gal*p*	59, 74, 87, 101, 118, 129, 143, 161, 202, 217	3.74	0.27	4.82
1,4,5-Me_3_-Gal*p*	1,4-Gal*p*	59, 71, 87, 99, 118, 157, 173, 188, 203	2.86	0.40	
2,4-Me_2_-Gal*p*	1,3,6-Gal*p*	87, 99, 115, 127, 142, 159, 217, 261	1.69	2.36	4.53
2,4-Me_2_-Rha*p*	1,3-Rha*p*	59, 72, 89, 101, 118, 131, 141, 160, 173, 187, 202	1.74		
3-Me-Rha*p*	1,2,4-Rha*p*	59, 74, 88, 101, 130, 143, 160, 171, 190, 203, 232	3.93	4.80	
2,3,5-Me_3_-Ara*f*	T-Ara*f*	59, 71, 87, 102, 113, 118, 129, 145	0.86		
2,3-Me_2_-Ara*f*	1,5-Ara*f*	59, 71, 87, 102, 118, 129, 173, 189	3.92		

**Table 3:** ^1^H/^13^C chemical shifts in DFIP-A3-1 from HSQC data.

Sugar Residues		Chemical Shifts, *δ* (ppm)					
		H-1/C-1	H-2/C-2	H-3/C-3	H-4/C-4	H-5/C-5	Ha, Hb-6/C-6
(A)	T-β-Gal*p*-(1→	4.49/102.4	3.45/72.9	3.59/73.0	3.89/69.7	3.65/75.9	3.72, 3.64/61.5
(B)	→6)-β-Gal*p*-(1→	4.45/103.7	3.56/71.8	3.67/72.9	3.96/69.4	3.81/73.6	3.96, 3.67/67.4
(C)	→3)-β-Gal*p*-(1→	4.68/103.9	3.83/73.2	3.85/81.7	4.23/70.3	3.70/74.2	3.81, 3.76/61.1
(D)	→4)-β-Gal*p*-(1→	4.58/104.4	3.61/73.0	3.70/74.2	4.10/76.8	3.64/76.0	3.72, 3.65/61.4
(E)	→3,6)-β-Gal*p*-(1→	4.69/103.8	3.83/69.8	3.85/81.8	4.26/70.3	3.96/76.4	3.96, 4.07/69.8
(F)	→2,4)-α-Rha*p*-(1→	5.21/98.7	4.09/76.9	3.81/69.7	4.01/80.2	3.81/68.8	1.27/17.3

**Table 4:** Quantitative real-time PCR primer sequences.

Gene	Primary Sequence
ACTIN	5′-GTGACGTTGACATCCGTAAAGA-3′
	5′-GTAACAGTCCGCCTAGAAGCAC-3′
NO	5′-CTTGGGCGATCCAGCTAATGT-3′
	5′-GTTGTCACAGTAATCACGAACGC-3′
IL-6	5′-ACAACCACGGCCTTCCCTAC-3′
	5′-GCACAACTCTTTTCTCATTTCCAC-3′
TNF-α	5′-CCCTCACACTCACAAACCACC-3′
	5′-CTTTGAGATCCATGCCGTTG-3′

## Data Availability

Data are contained within the article.

## Supplementary Materials

The following supporting information can be downloaded at: https://www.mdpi.com/article/10.3390/molecules31091461/s1, Figure S1: (a) Elution profile of FIP-A2-III-1 by preparative liquid chromatography. (b) Elution profile of DFIP-A3-1 and DFIP-A3-2 by preparative liquid chromatography; Figure S2: HPGPC-ELSD chromatogram of FIP-A2-III-1, FIP-A3, DFIP-A2-III-1, DFIP-A3-1 and DFIP-A3-2; Figure S3: In Vitro Immunomodulatory Screening of FIP-A to FIP-E. ($\bar{x}$ ± s; n = 3) * *p* < 0.05, ** *p* < 0.01, *** *p* < 0.001 vs. the control and ns for not statistically significant.

## Abbreviations

FIP	Folium isatidis polysaccharide
TFA	trifluoroacetic acid
HPGPC-ELSD	high performance gel permeation chromatography—evaporative light scattering detector
HPSEC-MALLS-RID	high performance size exclusion chromatography with multi-angle laser light scattering and refractive index detector
GC-MS	gas chromatography-mass spectrometry
SEM	scanning electron microscope
AFM	atomic force microscopy
*M*_w_	molecular weight
FT-IR	fourier transform infrared
RIPA buffer	Radioimmunoprecipitation assay buffer
SDS-PAGE	sodium dodecyl-sulfate polyacrylamide gel electrophoresis
PVDF	polyvinylidene fluoride
TBST	tris-buffered saline with Tween-20
LPS	lipopolysaccharide
TLR4	toll-like receptor 4
qRT-PCR	Quantitative reverse-transcription PCR
NF-κB	nuclear factor kappaB
