# Supplementary material for: Acid Degradation, Structure Characterization of a Novel Polysaccharide from Leaves of Isatis indigotica Fort. with Immunomodulatory Activity
Source: Molecules. 2026 Apr 28;31(9):1461. doi: 10.3390/molecules31091461 (PMC13164619; doi:10.3390/molecules31091461)
Supplement: Supplementary file 1 [file molecules-31-01461-s001.zip › molecules-4224890-supplementary.pdf]

Acid degradation，structure characterization of a novel polysaccharide from leaves of *Isatis indigotica* Fort. with immunomodulatory activity

Yu Shen [1], Xuefeng Wang [1], Huiming Zhang [2], Yuliang Wang [1], Zheng Wang [1], Yiying Zhang [3,*] and Hongbo Zhao [4,*]

1 College of Pharmacy, Jiamusi University, Jiamusi 154007, China; shenyu@jmsu.edu.cn (Y.S.); 238163035@stu.jmsu.edu.cn (X.W.); wangyuliang@jmsu.edu.cn (Y.W.); 258073057@stu.jmsu.edu.cn (Z.W.)

2 College of Basic Medical Sciences, Jiamusi University, Jiamusi 154007, China; zhmjms@jmsu.edu.cn (H.Z.)

3 Department of Epidemiology and Biostatistics, School of Public Health, Jiamusi University, Jiamusi 154007, China

4 College of Rehabilitation Medicine, Jiamusi University, Jiamusi 154007, China

* Correspondence: zhangyiying@jmsu.edu.cn (Y.Z); zhaohongbo@jmsu.edu.cn (H.Z)

Figure Caption

Figure S1. (a) Elution profile of FIP-A2-III-1 by preparative liquid chromatography. (b) Elution profile of DFIP-A3-1 and DFIP-A3-2 by preparative liquid chromatography.

Figure S2. HPGPC-ELSD chromatogram of FIP-A2-III-1, FIP-A3, DFIP-A2-III-1, DFIP -A3-1 and DFIP -A3-2.

Figure S3. In vitro immunomodulatory screening of FIP-A to FIP-E. ($\bar{x} \pm$ s; n = 3) * $p < 0.05$, ** $p < 0.01$, *** $p < 0.001$ vs. the control and ns for not statistically significant.

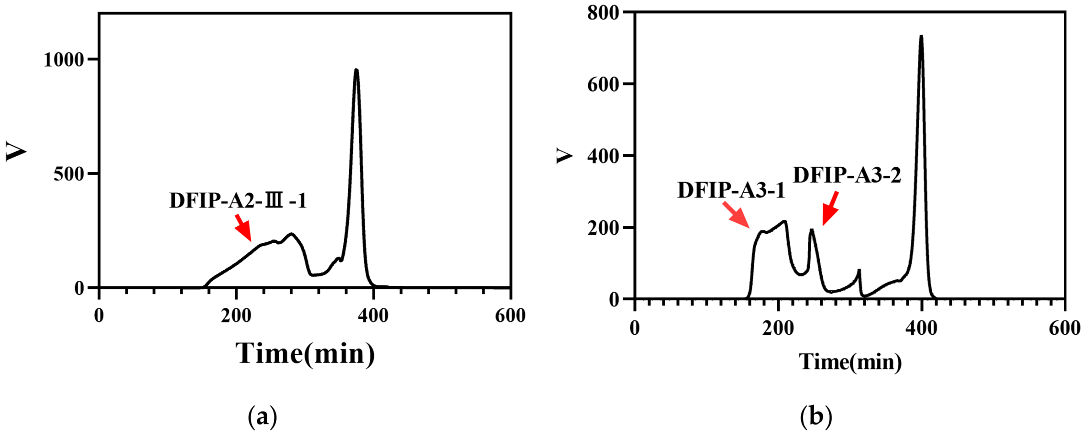

Figure S1.

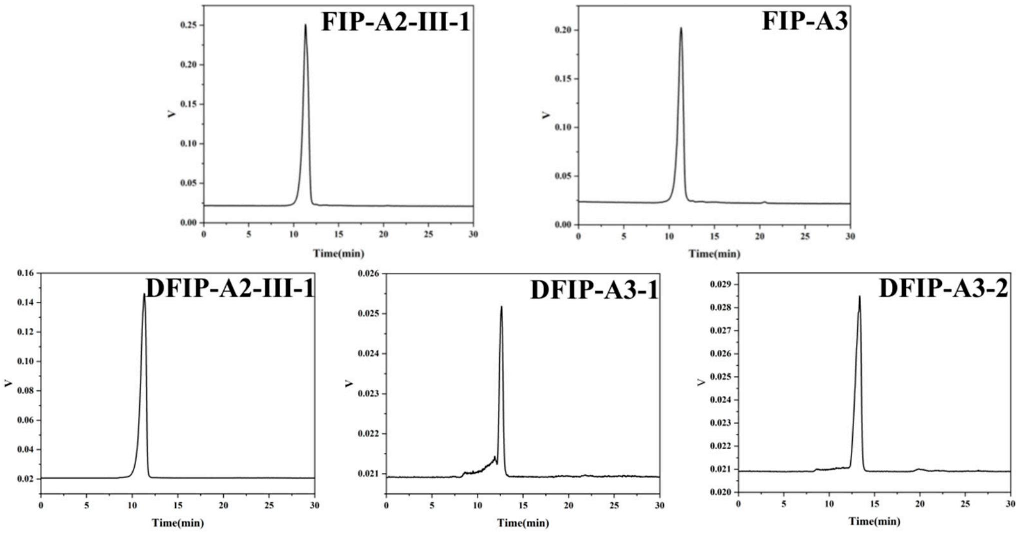

Figure S2.

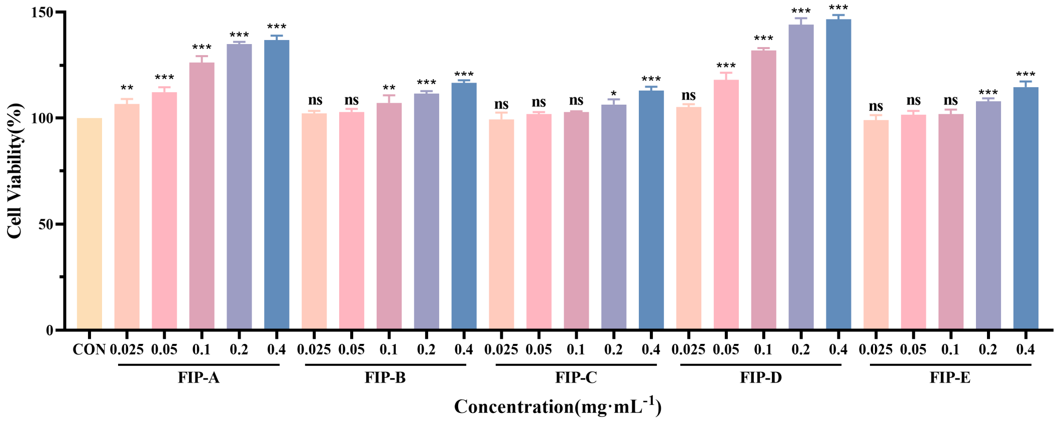

150
100
50
0
Cell Viability(%)
CON
0.025 0.05 0.1 0.2 0.4
FIP-A
FIP-B
FIP-C
FIP-D
FIP-E
Concentration(mg·mL-1)
**
***
ns
*

Figure S3.
